# Tree growth response and adaptation to climate change and climate extremes: From canopy to stem

**DOI:** 10.1111/jipb.70145

**Published:** 2026-01-20

**Authors:** Feiyu Yang, Leyao Zhu, Jiahao Cao, Fei Yang, Borbála Codogno, Qianqian Ma, Hanxue Liang, Wenjin Wang, Jian‐Guo Huang

**Affiliations:** ^1^ State Key Laboratory for Vegetation Structure, Function and Construction (VegLab), MOE Key Laboratory of Biosystems Homeostasis and Protection, College of Life Sciences Zhejiang University Hangzhou 310058 China; ^2^ Guangdong Provincial Key Laboratory of Applied Botany, South China Botanical Garden the Chinese Academy of Sciences Guangzhou 510650 China; ^3^ Institute of Loess Plateau Shanxi University Taiyuan 030006 China; ^4^ Swiss Federal Institute for Forest Snow and Landscape Research (WSL) Birmensdorf 8903 Switzerland

**Keywords:** adaptation, annual stem growth, canopy phenology, climate change, climate extremes, intra‐annual wood formation, response, tree growth

## Abstract

Ongoing climate warming has altered precipitation patterns and increased the frequency and intensity of climate extremes such as droughts, heatwaves, floods, and frosts. These changes have significantly influenced tree growth and development processes, including canopy phenology, intra‐annual wood formation dynamics, and annual stem growth. However, these processes are affected by various climatic factors, and their responses are highly species‐specific and vary across temporal and spatial scales. Beyond these rapid growth responses, trees may also undergo long‐term genetic adaptation to climate change. This review synthesizes how canopy phenology, intra‐annual wood formation dynamics, and annual stem growth respond to climate change and climate extremes. We summarize the response and adaptation of these growth processes to various climatic drivers and highlight the interactions among them in determining tree growth. Concepts and mechanisms of rapid response and heritable genetic adaptation in trees under climate change are also reviewed. We identify the key knowledge gaps in tree growth response and adaptation, such as integrative multiple organ and growth process monitoring and genetic‐level studies, which are critical to further improve our understanding of tree growth to support sustainable forest management and enhance forest carbon storage under ongoing climate warming.

## Graphical abstract

This review synthesizes the responses and adaptations of tree growth, including canopy phenology, intra‐annual wood formation dynamics, and annual stem growth, to climate change and climate extremes. It highlights key knowledge gaps for future research to support sustainable forest management and enhance forest carbon storage under ongoing climate warming.

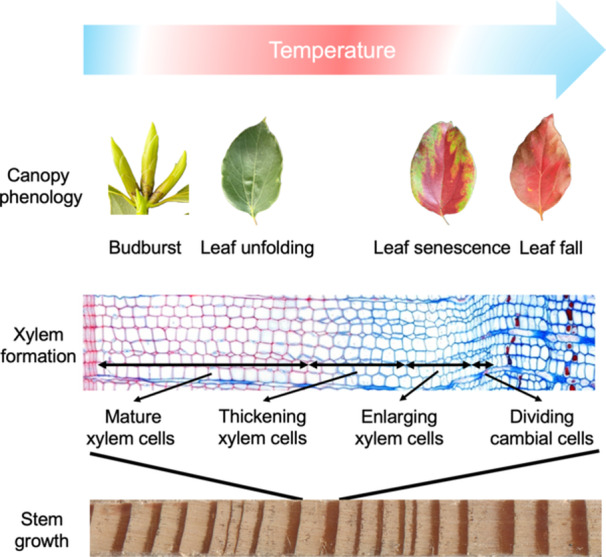

## INTRODUCTION

Forests, as the largest terrestrial carbon sink, play a critical role in the global carbon, water, and energy cycles and are essential for mitigating climate change (Nolan et al., 2021). In addition, forests provide a wide range of ecological, economic, and cultural services that support human societies (Bonan, 2008). However, ongoing climate warming and changes in precipitation over the past century (Masson‐Delmotte et al., 2021), together with more frequent and intense climate extremes such as droughts, heatwaves, floods, and frosts (Liu et al., 2018; AghaKouchak et al., 2020; Fischer et al., 2025; Martinez‐Villalobos et al., 2025), have profoundly impacted the structure and function of forest ecosystems (Trumbore et al., 2015; Seidl et al., 2017).

In recent years, increasing attention has been paid to forest responses to climate change and climate extremes, revealing diverse growth responses across regions and species. For example, rising temperatures in cold, high‐latitude regions may enhance forest growth, while in arid or semi‐arid areas, warming tends to exacerbate water stress, suppress growth, and even trigger large‐scale tree mortality (Tei et al., 2017; Yan et al., 2024). Compared with average climate conditions, climate extremes often have more severe and complex impacts on forest ecosystems, which may lead to acute physiological damage and prolonged recovery times (Frank et al., 2015; Lamichhane, 2021). Although forest growth may have initially benefited from the CO_2_ fertilization effect in the early stages of warming, recent studies indicate a widespread slowdown in forest growth, increasing uncertainty in global carbon sink capacity (Zhang et al., 2022). Understanding how forests respond and adapt to climate change and climate extremes is thus crucial for accurately evaluating their carbon sequestration potential, modeling the terrestrial carbon cycle, and developing effective forest management strategies (Seidl et al., 2017; Gao et al., 2022).

**Figure 1:**
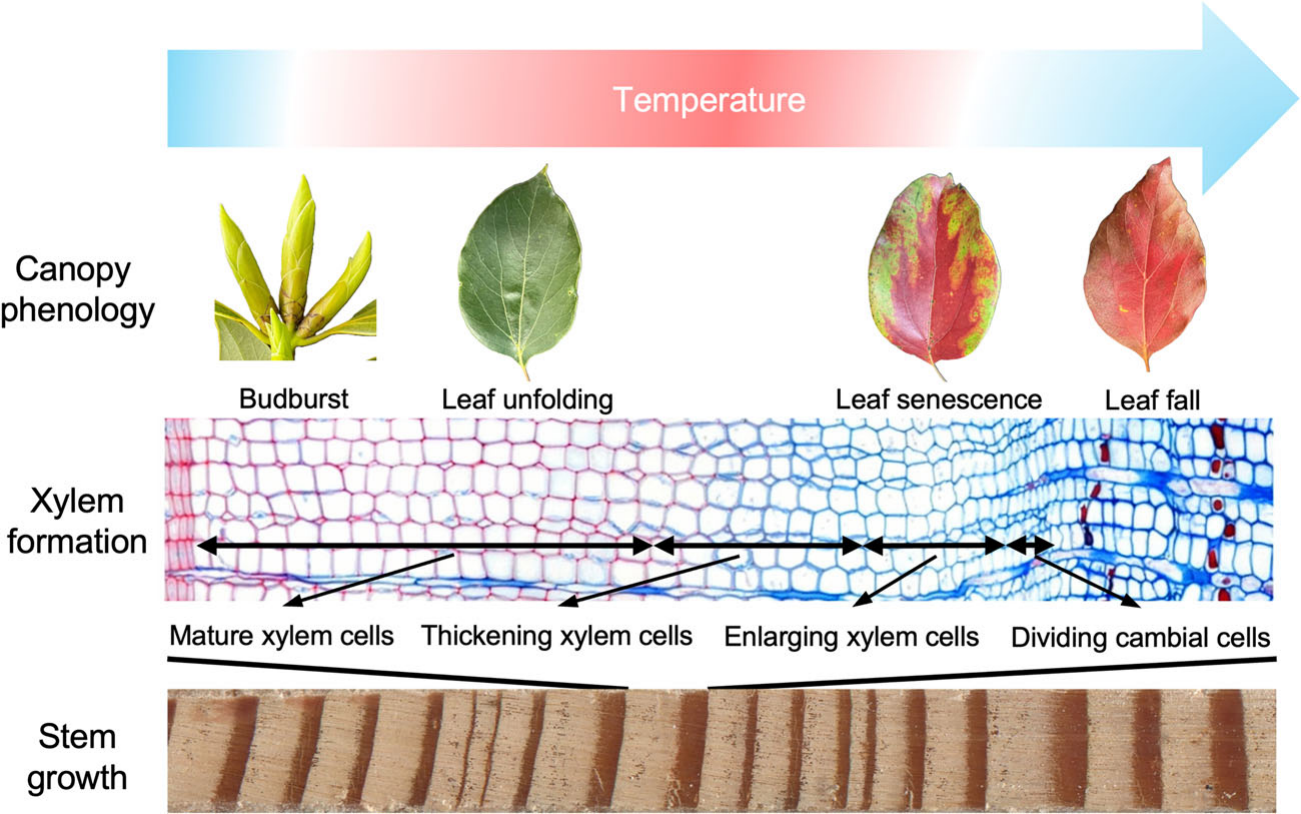
Schematic of tree growth and development processes Intra‐annual dynamics of canopy phenology and xylem formation during the growing season. Xylem formation was monitored using microcore samples (cell‐level observations). This sample was collected during the stem growing season (i.e., the period of tree‐ring formation), when all stages of wood formation were present. As the progress of leaf phenology and xylem formation varies among species and regions, the schematic illustrates the typical stages of canopy and xylem development. The directions of xylem formation and stem growth are shown from the pith to the bark (left to right). Cambial cells differentiate into xylem cells toward the pith. The color gradient from blue to red represents the annual temperature cycle in the Northern Hemisphere, transitioning from cold to hot and back to cold, which generally drives the timing of canopy phenology and stem growth.

Tree growth comprises both primary growth (e.g., leaf development) and secondary growth (e.g., stem growth), which jointly determine aboveground biomass production and long‐term carbon accumulation (Cuny et al., 2015; Figure 1). Ongoing climate warming and increasing climate extremes have affected both primary and secondary growth, ultimately influencing carbon allocation strategies and forest productivity (Bonan, 2008). However, these growth processes are influenced by multiple climatic factors and vary greatly across species and regions. Moreover, leaf development provides the materials and energy required for secondary growth and can therefore directly influence secondary growth (Körner et al., 2023). Many studies focused on primary or secondary growth response to climate change and climate extremes separately, while overlooking their linkages. A better understanding of their interactions and how climate regulates these linkages is essential for accurately modeling forest growth.

Responses are rapid, short‐term adjustments to climate variability, whereas adaptations involve long‐term, heritable evolutionary changes under sustained climate change. As climate warming is driving fast and dramatic changes in environmental conditions, trees are expected to undergo long‐term adaptations to maintain growth and reproduction. Failure to adapt may increase mortality and even increase the risk of extinction, ultimately altering forest structure and reducing biodiversity (Boonman et al., 2024; Chakraborty et al., 2024). Changes in canopy phenology and stem growth are the earliest observable responses of trees to climate change and may act as precursors to adaptive processes. These rapid responses are primarily driven by changes in gene expression triggered by climate fluctuations, which are often temporary and may occur within a single year. For example, leaf phenology is regulated by the expression of a gene network that is sensitive to temperature and photoperiod (Maurya et al., 2018). In contrast, mutations provide the potential for heritable adaptations to prolonged climate change (Yona et al., 2015). Clarification of genetic‐level changes in tree growth is essential for revealing the mechanisms underlying tree adaptation to climate change, thereby reducing potential risks to their survival under future conditions. Furthermore, trees may migrate to more suitable habitats, mainly through seed dispersal, representing an adaptive strategy under climate change (Dyderski et al., 2025). Many tree species have shifted their distributions toward higher altitudes and latitudes due to global warming (Rotbarth et al., 2023; Lu et al., 2025). Understanding and predicting these range shifts are important for maintaining and enhancing forest ecosystem functioning and carbon storage (Chakraborty et al., 2024).

This review synthesizes current knowledge on the response and adaptation of tree growth to climate change and climate extremes. We specifically focus on canopy phenology and stem growth, considering their responses across temporal, spatial, and species‐level scales, as well as the physiological mechanisms underlying these processes. The objectives are to (i) summarize the responses of canopy phenology, annual tree radial growth, and intra‐annual wood formation to climatic drivers and climate extremes; (ii) examine the relationships among these growth processes and the factors regulating them; (iii) discuss the adaptation of tree growth under climate change; and (iv) highlight how to improve the modeling and prediction of tree growth for future climates. This review contributes to a comprehensive understanding of tree growth dynamics under climate change, provides support for enhancing forest management and carbon sequestration, and proposes key directions for future research.

## CANOPY PHENOLOGY RESPONSE TO CLIMATE CHANGE

Canopy phenology, a key indicator of tree growth dynamics, describes the timing of annually recurring developmental stages such as budburst, leaf unfolding, flowering, fruiting, leaf senescence, and leaf fall (Schwartz, 2024). Climate change has led to substantial changes in canopy phenology worldwide. These changes have broad impacts on tree growth, carbon and water cycles, and species interactions (Piao et al., 2019a; Menzel et al., 2020; Lang et al., 2024).

### Canopy phenology monitoring

Canopy phenology has been documented primarily through multiple *in situ* observation networks, such as the Pan European Phenology network (PEP725) (Templ et al., 2018), the USA National Phenology Network (USANPN) (Schwartz et al., 2012), the Russian Chronicles of Nature Network (RCNN) (Ovaskainen et al., 2020), and the China Phenological Observation Network (CPON) (Zhu et al., 2024). These large‐scale, long‐term databases, covering multiple phenological stages and tree species, provide the most accurate records and form the foundation for research in tree phenology. In addition, herbarium specimen records also serve as an important source of phenological information (Jones and Daehler, 2018). Unlike phenology monitoring networks based on continuous observations of the same individuals or sites, herbarium records are usually collected irregularly across species, sites, and years (Park and Schwartz, 2015). Herbarium‐based phenological studies predominantly focus on flowering, as flowering is the most consistently recorded and reliably identified in preserved specimens, whereas other stages of leaf development are less well represented (Jones and Daehler, 2018). These records provide sparse observations across a wide range of species and spatial scales, enabling assessments of phenological change at community and regional levels. For example, an analysis of 19,328 flowering herbarium specimens collected during 1951–2009, retrieved from digital herbarium archives, showed that flowering advanced with increasing March temperatures (Park and Schwartz, 2015).

However, *in situ* phenological observations are sparsely distributed, cover different time spans, and lack a unified definition for phenological events, which largely reduces data consistency and limits data integration across regions and species (Piao et al., 2019a). In contrast, advances in remote sensing over the past decades have provided global coverage and greatly improved the consistency and accuracy of canopy phenological monitoring, which is now widely applied in large‐scale vegetation phenology research (Gong et al., 2024). Vegetation indices such as the Normalized Difference Vegetation Index (NDVI) and the Enhanced Vegetation Index (EVI), derived from the Advanced Very High Resolution Radiometer (AVHRR) (Wang et al., 2019), the Moderate Resolution Imaging Spectroradiometer (MODIS) (Friedl and Sulla‐Menashe, 2022), the Landsat satellite series (Kowalski et al., 2020), and the Sentinel missions (Misra et al., 2020), provide relatively continuous records at multiple temporal and spatial resolutions, enabling large‐scale assessment of canopy phenology (Gong et al., 2024). Automated camera networks such as the Phenology Camera (PhenoCam) Network also allow high‐frequency, site‐level monitoring of canopy phenology across diverse ecosystems (Richardson et al., 2018). PhenoCam is ideal for localized studies, as it provides more accurate data than remote sensing (Richardson et al., 2018). Overall, multi‐source data provide critical observations for understanding tree growth dynamics and their responses to climate change.

Although remote sensing has greatly advanced research in canopy phenology, several limitations remain. While the state‐of‐the‐art satellites and sensors offer high spatial resolution, such as Sentinel‐2 at 10 m resolution, their temporal coverage is limited to about 10 years and may not be suitable for long‐term research. In general, data sets with higher spatial resolution provide shorter observational records, and so, choosing the most appropriate data sets is critical for phenology studies. Although remote sensing provides global coverage and continuous monitoring of canopy phenology, it only coarsely estimates phenological events from vegetation index thresholds or their rates of change (Zeng et al., 2020). For example, the MODIS Land Cover Dynamics (MCD12Q2) data product provides seven stages of global land surface phenology, including Greenup, Mid‐Greenup, Maturity, Peak, Senescence, Mid‐Greendown, and Dormancy, derived from fixed EVI thresholds (i.e., 15%, 50%, 90%, and 100% of the maximum EVI) (Friedl and Sulla‐Menashe, 2022). However, these metrics do not necessarily reflect actual phenological events compared with *in situ* observations. Moreover, as remote sensing provides gridded phenology, estimates may be influenced by the variability of land cover type, leading to deviations from true phenological stages (Zeng et al., 2020). Therefore, the integration of remote sensing and *in situ* observations, which enables cross‐validation of phenology estimates, is essential for robust and comprehensive monitoring of canopy phenology and for assessing their responses to climate change (Gong et al., 2024).

### Changes in canopy phenology and climatic drivers

#### Vegetative phenology

**Figure 2:**
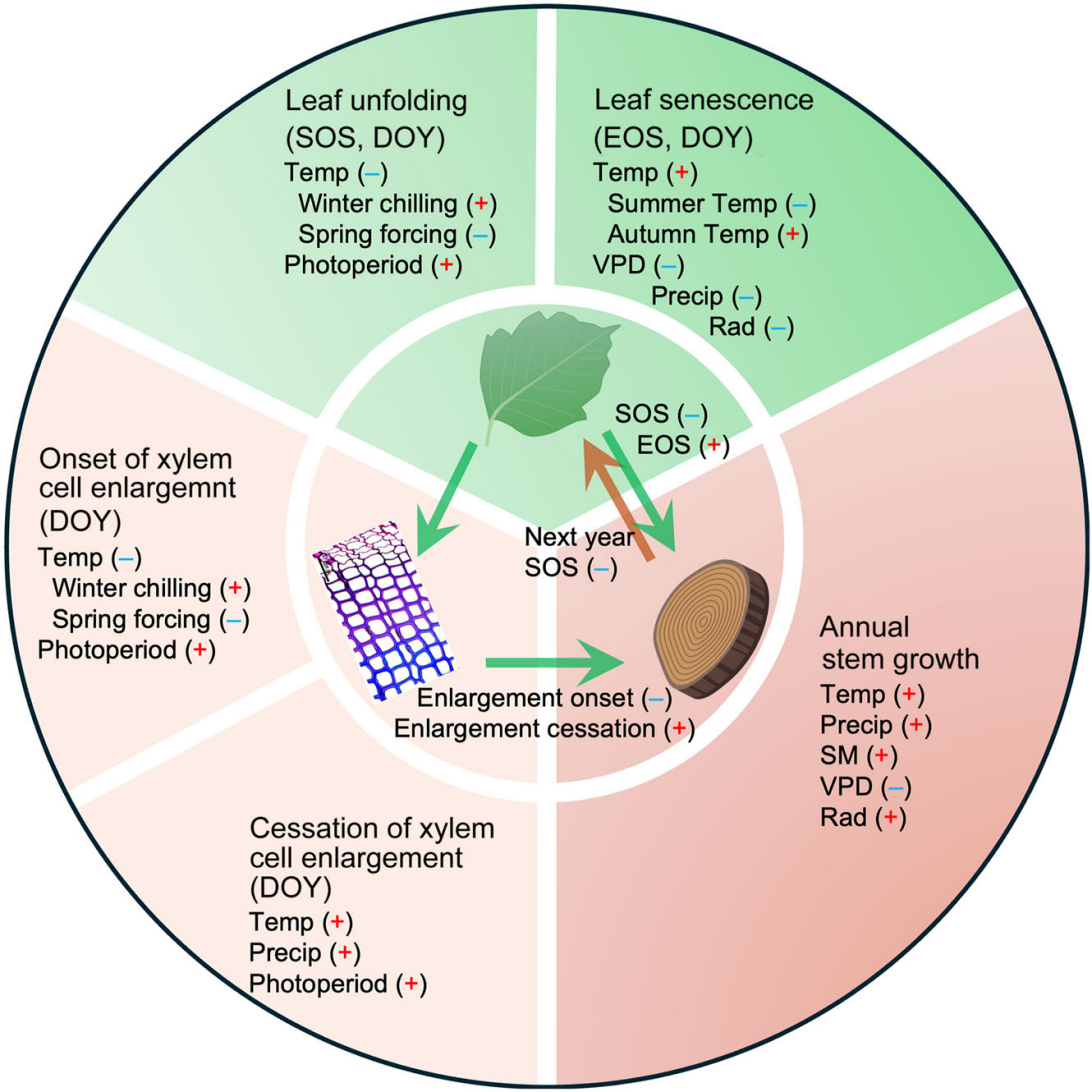
Summary of climatic factors influencing key stages of leaf phenology and xylem formation and annual stem growth, together with the interactions among these growth processes The onset of leaf unfolding (day of year, DOY) and leaf senescence (DOY) defines the leaf‐based growing season, whereas the onset and cessation of xylem cell enlargement (DOY) define the wood‐based growing season. Stem growth reflects the amount of annual stem growth. The symbols in brackets denote the direction of influence (“+” indicates a positive effect; “−” indicates a negative effect). For example, a higher annual mean temperature may lead to a smaller DOY (negative effect, i.e., advancement) of the onset of xylem cell enlargement. Temp: annual mean temperature; Precip: annual total precipitation; VPD: annual mean vapor pressure deficit; SM: annual mean soil moisture; Rad: annual mean shortwave radiation; and Photoperiod: daylength. Interactions among growth processes. Earlier leaf unfolding (SOS) and later leaf senescence (EOS) may prolong the leaf‐based growing season and promote stem growth. Conversely, enhanced stem growth may lead to an earlier SOS in the next year. Earlier onset and later cessation of xylem enlargement are generally considered to extend the duration of xylem cell production, thereby potentially enhancing total stem growth. The relationship between leaf phenology and intra‐annual wood formation dynamics cannot be easily generalized, as the sequence of these processes varies among species. We acknowledge that these interactions are summarized from large‐scale studies and may not always be consistent across different sites or species.

Vegetative phenology refers to the seasonal development of vegetative parts such as leaves, especially the timing of leaf unfolding and senescence, which typically marks the start (SOS) and end of the growing season (EOS) in trees and directly influences carbon assimilation and ecosystem productivity (Körner et al., 2023). Both *in situ* and satellite observations show an overall earlier leaf unfolding and later leaf senescence, resulting in a prolonged growing season across the extratropical Northern Hemisphere (> 30°N) over the past 50 years (Menzel et al., 2006; Piao et al., 2019a). Specifically, satellite observations from 1982 to 2011 indicated that about 75% of vegetation sites showed an advance in SOS, at a rate of 2.1 d per decade, while about 65% of sites showed a delayed trend in EOS, at 1.8 d per decade (Piao et al., 2019a). Similarly, *in situ* observations from the PEP725 network indicated that about 93% of sites experienced an advance in SOS at a rate of 4.2 d per decade, whereas about 50% of sites showed a delay in EOS of 0.1 d per decade from 1982 to 2011 (Piao et al., 2019a). As a result, this extended growing season has lengthened the duration of photosynthetic activity, thereby contributing to greater productivity and biomass accumulation in many regions (Keenan et al., 2014). Different phenological stages respond to climate change through multiple mechanisms driven by various climatic factors such as temperature and photoperiod (Figure 2). Understanding these mechanisms and their interactions with climatic factors is essential for predicting future phenological changes under ongoing climate warming.

Temperature is the most important factor that influences spring leaf phenology. In general, the timing of spring phenology is regulated by a temperature‐derived dormancy mechanism. During winter, accumulated cold (chilling) is required to transition from endodormancy to ecodormancy, followed by sufficient heat accumulation (forcing) in spring to initiate the onset of spring phenology (Hänninen, 1990). Although spring phenology is highly sensitive to temperature changes, the direction and magnitude of these responses can vary depending on the timing of warming. Spring warming typically accelerates the heat accumulation to terminate dormancy early, thereby advancing the spring phenology (Yu et al., 2010). In contrast, increases in winter temperatures can reduce cold accumulation, leading to insufficient chilling for endodormancy release and ultimately delaying spring phenology (Fu et al., 2015). This winter warming has slowed the advance of spring phenology observed in recent decades (Fu et al., 2015; Asse et al., 2018). Consequently, the net effect of warming on spring phenology depends on the balance between enhanced spring forcing and reduced winter chilling. Due to global warming, winter temperatures have been rising faster than spring temperatures. This raises the question of whether advances in spring leaf phenology may slow down or even reverse as warmer winters lead to insufficient winter chilling.

In addition, the responses of spring phenology to warming vary among species and across regions. For example, species that leaf out earlier tend to have lower chilling requirements for spring phenology (Fu et al., 2012). The chilling requirement for leaf unfolding is higher in temperate regions than in subtropical regions, whereas subtropical regions require greater forcing temperatures compared to temperate regions (Xu et al., 2021; Yu et al., 2023). Moreover, the effects of temperature change are asymmetric across diurnal scales (Meng et al., 2020). The relationships between SOS and nighttime temperature and between SOS and daytime temperature are opposite in direction (Meng et al., 2020). Although an overall advancing trend was observed from the 1970s to 2000 in response to global warming (Menzel et al., 2006), satellite‐derived spring phenology across the Northern Hemisphere (> 30°N) suggests that the earlier trends of spring phenology advancement have weakened since 1998, during the period of the global warming hiatus (Wang et al., 2019), and are even projected to reverse after 2060 (Mo et al., 2024). These findings highlight that trends in spring phenology are highly variable over time, which increases the uncertainty in predicting future spring phenology under global warming. Therefore, understanding the underlying mechanisms and region‐specific responses, together with continuous large‐scale monitoring, is essential for improving the modeling and prediction of future spring phenology.

Photoperiod also plays a critical role in regulating spring phenology. Photoperiod varies with geographical latitudes but remains unchanged among years for a given location (Way and Montgomery, 2015), thus providing a more common and stable growth signal compared with temperatures (Körner and Basler, 2010). Moreover, photoperiod may adjust the efficiency of both chilling and forcing processes (Meng et al., 2021). Photoperiod can delay early leaf‐out induced by temperature fluctuations, thereby moderating spring advancement under warming and reducing the risk of late spring frost damage (Fu et al., 2019; Meng et al., 2021). During a cold spring, photoperiod can advance delayed spring phenology toward an optimal timing of solar radiation that favors photosynthesis (Fu et al., 2019). This regulatory effect is particularly pronounced in high‐elevation regions, where temperature is more variable, and photoperiod provides a more reliable signal for spring phenology (Basler and Korner, 2014). Based on these fundamental mechanisms and their associated climatic factors, such as temperature and photoperiod, numerous process‐based models (e.g., photothermal‐time model) and empirical models (e.g., machine learning) have been developed to simulate spring phenology (Basler, 2016; Hufkens et al., 2018; Garnot et al., 2025).

Global warming has also led to a general delay in autumn phenology, but the trends are more variable than those of spring phenology (Piao et al., 2019a; Hassan et al., 2023). The mechanisms underlying autumn phenology and the associated climatic factors are also more complex and less well understood (Zani et al., 2020), resulting in relatively poor performance in modeling autumn phenology (Garnot et al., 2025). Temperature is considered the primary driver of autumn leaf senescence (Zohner et al., 2023). Warmer autumn temperatures delay leaf yellowing, the most obvious event of senescence in deciduous trees, by slowing chlorophyll degradation (Matos et al., 2012). However, recent research has shown that warmer temperatures after the summer solstice only slow the progression of leaf senescence, while warmer temperatures before the summer solstice advance the onset of leaf senescence by increasing vegetation activity, which suggests that warming during different seasons has contrasting effects on leaf senescence (Zohner et al., 2023). This was further supported by the improved accuracy of the model of leaf senescence that incorporates productivity during the growing season (Zani et al., 2020), as warmer summer temperatures and higher precipitation accelerate productivity saturation, leading to advanced autumn senescence due to sink limitation (Zani et al., 2020). Similarly, strong shortwave radiation can enhance photosynthesis and productivity, ultimately leading to earlier leaf senescence (Wu et al., 2021). These findings emphasize that both climatic factors and carbon dynamics must be considered to accurately model and predict autumn phenology.

While temperature is the primary driver of leaf phenology, water availability also plays an important role. In water‐limited regions, precipitation has been shown to advance spring phenology (Shen et al., 2015). Precipitation can also have contrasting effects on leaf senescence. Higher precipitation may advance leaf senescence by accelerating productivity (Zani et al., 2020), whereas drought tends to trigger leaf senescence to preserve water (Wu et al., 2022). These findings suggest the presence of a threshold in water availability that regulates the timing of leaf senescence, balancing the trade‐off between productivity and water conservation. Despite the complex effects of water on leaf phenology, water availability has not been considered in most phenology models (Basler, 2016), and the extent to which water availability may affect leaf phenology remains unquantified.

#### Reproductive phenology

Reproductive phenology, including flowering and fruiting, plays a critical role in determining reproductive success and maintaining ecosystem structure, function, and stability (Elzinga et al., 2007; Franks, 2015; Guo et al., 2023), but it has received less attention compared to leaf phenology (Delpierre et al., 2016). Global warming has generally led to earlier flowering and fruiting (Bock et al., 2014; Ma et al., 2022). Similar to spring phenology, flowering is highly sensitive to temperature, which is regulated by forcing and chilling (Guo et al., 2023). The magnitude of flowering responses to warming also differs across species and environments (Collins et al., 2021). For instance, spring‐flowering species typically show a stronger response to warming than summer‐flowering species, as the latter rely more heavily on photoperiod for phenological regulation (Aldridge et al., 2011). Several studies have examined the interval between leaf‐out and flowering. Based on phenology records from the CPON, intervals between leaf‐out and flowering are generally stable, although some variation occurs, with shorter intervals in leaf‐first species and longer intervals in flower‐first species, as flowering is generally more sensitive to temperature than leaf‐out (Guo et al., 2023). In addition, phenology records from PEP725 revealed that spring warming tends to shorten the interval between leaf‐out and flowering, whereas winter warming tends to lengthen this interval (Ma et al., 2021).

Fruit development begins with flowering and continues until the fruit matures, which is largely regulated by temperature, forcing accumulation, and precipitation during this period (Ma et al., 2022). Variation in this fruit development period can influence the quantity and quality of fruit and seed, ultimately affecting overall reproductive success (Sandor et al., 2021; Ma et al., 2022). For example, a longer fruit development period generally results in larger fruit size in peach (Wert et al., 2009). Although both flowering and fruiting tend to advance under global warming, the degree of advancement differs between them, resulting in species‐specific variation in the fruit development period (Ma et al., 2022). Overall, rising temperatures may shorten the fruit development period, whereas greater forcing requirements tend to prolong it (Ma et al., 2022). A better understanding of reproductive phenology is essential for assessing tree fitness, inter‐ and intra‐specific interactions, and for understanding how trees allocate resources among different organs across different life stages (Sandor et al., 2021; Guo et al., 2023).

### Canopy phenology response to climate extremes

Global warming has increased the risk of late spring frost due to the earlier onset of spring phenology (Liu et al., 2018). Late spring frost, also known as “false spring”, has been widely documented to cause leaf and flower damage (Inouye, 2008; Hufkens et al., 2012; Vitasse et al., 2025), leading to substantial reductions in tree growth and productivity and resulting in ecological impacts and economic losses (Chamberlain et al., 2019). The period following budburst is most sensitive to frost, while frost tolerance gradually increases as leaves reach maturity (Chamberlain et al., 2019). Daily minimum temperatures falling below approximately −2.2°C after budburst are generally considered to cause damage to new leaf tissue (Schwartz et al., 2006). The false spring index (FSI), calculated as the difference between the day of the last spring freeze (often defined as < −2.2°C) and the day of budburst, is widely used to evaluate the risk of frost damage (Chamberlain et al., 2019). However, this index overlooks differences among species, which can lead to inaccurate assessments of frost risk (Chamberlain et al., 2019).

The risk of frost varies widely across both species and regions. Species with spring phenology highly sensitive to warming face a higher risk of frost damage, and the risks are becoming more frequent and severe in maritime and coastal regions of Europe compared with continental regions (Ma et al., 2019). Species have evolved diverse strategies to avoid frost damage. Although temperate tree species differ in their leaf‐out dates, they tend to converge on leaf‐out dates when the risk of frost is minimal (Lenz et al., 2016). Trees also tend to avoid periods of high frost risk by delaying budburst or speeding up leaf development to minimize potential frost damage (Chamberlain et al., 2019). Moreover, the frost experiment showed that flowers are more susceptible to frost than leaves, as their rapidly differentiating and actively growing tissues are more vulnerable (CaraDonna and Bain, 2016). Long‐lived plants also tend to prioritize the protection of vegetative structures over short‐lived reproductive organs, thereby ensuring future survival and opportunities for subsequent reproduction (CaraDonna and Bain, 2016). Further long‐term field monitoring and laboratory experiments are needed to measure frost thresholds across species and phenological stages, and understanding how trees avoid and tolerate frost is essential for better predicting and preventing potential frost damage (Chamberlain et al., 2019; Kirchhof et al., 2025).

Global warming increases the frequency and intensity of droughts. However, their impacts on canopy phenology remain poorly understood. *In situ* and satellite observations revealed that drought‐induced water limitation can advance leaf senescence, as trees shed leaves to reduce water loss and maintain hydraulic balance (Wu et al., 2022). Droughts also substantially delayed spring phenology in the following year, primarily due to reduced soil moisture in spring, suggesting that future spring phenology advancement may be weaker than previously expected under increasing drought (Liu et al., 2025a). These effects are not captured by current phenology models (Liu et al., 2025a), highlighting the need to improve our understanding of drought impacts on canopy phenology for future incorporation into phenology models.

## TREE RADIAL GROWTH RESPONSE TO CLIMATE CHANGE

Tree stems serve as the primary reservoir of carbon in forests and play a crucial role in long‐term carbon storage (Fatichi et al., 2014; Cuny et al., 2015; Jiang et al., 2020). Thus, variations in stem radial growth can significantly affect forest carbon stocks (Babst et al., 2014; Etzold et al., 2022). Stem growth is highly sensitive to climate change and climate extremes. Although many studies have demonstrated that climate significantly affects stem radial growth (Tei et al., 2017; Babst et al., 2019; Yang et al., 2023; Li et al., 2024), uncertainty remains regarding the magnitude and temporal and spatial variability of these responses (D'Arrigo et al., 2008; D'Orangeville et al., 2018; Seftigen et al., 2018). A comprehensive understanding of how stem radial growth responds to global warming is therefore essential for predicting terrestrial carbon cycling and developing effective forest management strategies (Anderegg et al., 2020).

### Tree radial growth monitoring

Tree rings provide a clear and continuous record of annual variation in stem growth and are widely used to assess the effects of climate on forest growth. In particular, tree‐ring width is a direct measure of stem radial growth, as it offers high temporal resolution, can be accurately dated, is sensitive to climate variability, and has broad spatial coverage (Sheppard, 2010; Esper et al., 2016). Trees can live for several centuries or even millennia, during which they experience a wide range of climatic conditions, such as different temperatures and water conditions. In general, when climatic conditions are favorable for growth, trees form wider rings, whereas adverse conditions result in narrower rings. Therefore, tree‐ring width records the influence of climate on tree radial growth and has been used to investigate the responses of stem growth to climate change and climate extremes (Sheppard, 2010).

Tree‐ring samples are usually collected and analyzed following standard dendrochronological procedures (Fritts, 1976), including extraction of tree‐ring cores from trees, laboratory preparation (e.g., sanding), crossdating to ensure accurate temporal alignment of ring‐width series across samples, measurement of ring widths, and verification of the quality of crossdated ring‐width series. The crossdated ring‐width series should be detrended and standardized. In addition to environmental factors, tree growth is also influenced by intrinsic physiological factors such as tree age and size. As trees grow older and their stems become larger, the similar annual increment of biomass is spread over a larger circumference, causing ring width to gradually decrease from the pith toward the bark. To remove the decreasing trends in ring width associated with tree age and size, an appropriate fitting function should be applied (Sheppard, 2010). After detrending, the ring‐width series were standardized to produce ring‐width index time series, also known as chronologies.

In addition to field sampling, tree‐ring records can be obtained from open‐access databases. For example, the International Tree‐Ring Data Bank (ITRDB) collects tree‐ring measurements for over 200 tree species from more than 6,000 sites worldwide, serving as the world's largest repository of dendrochronological data (Zhao et al., 2019). These tree‐ring data serve as essential resources for investigating global forest growth dynamics, reconstructing paleoclimate, and assessing the impacts of climate change on forest ecosystems (Babst et al., 2017; Neukom et al., 2019; Zhao et al., 2019). For example, tree‐ring width represents one of the most important proxies for reconstructing temperature over the past two millennia (Neukom et al., 2019) and has been used to capture millennial‐scale streamflow variability (Liu et al., 2020; Chen et al., 2023). Additionally, tree‐ring width data have revealed that earthquake‐induced soil cracks and fractures facilitate deeper precipitation infiltration, potentially enhancing soil water and nutrient availability and increasing tree resilience to drought (Gao et al., 2024). Tree‐ring width data have also been used to reconstruct extreme events, such as droughts, heatwaves, frosts, and floods (details are provided in subsequent sections about climate extremes). Furthermore, tree‐ring width reveals the decoupling between carbon assimilation and wood production, highlighting that tree growth is often limited by sink activity rather than carbon availability (Cabon et al., 2022). In addition, other tree‐ring measurements such as wood density and stable isotopes also provide valuable insights into the relationships between tree growth and climate (Björklund et al., 2019; Cherubini et al., 2021). For instance, tree‐ring δ^18^O and δ^13^C measurements have been applied to reconstruct past precipitation at millennial scales (Liu et al., 2025b; Qin et al., 2025).

### Changes in tree radial growth and climatic drivers

Tree ring widths have been widely used to reconstruct historical climate because of their generally stable relationship with climate variability (Esper et al., 2016; Neukom et al., 2019). However, increasing evidence shows that this growth–climate relationship is shifting under ongoing climate warming (D'Arrigo et al., 2008; Babst et al., 2019; Li et al., 2024). Changes in the sensitivity of tree radial growth to climate may increase the uncertainty in climate reconstruction and projection of future tree radial growth (D'Arrigo et al., 2008).

Over the past century, water availability has increasingly become one of the most critical limiting factors of global tree radial growth (Babst et al., 2019). In high‐latitude regions of the Northern Hemisphere, temperature is the key climatic factor limiting tree growth (Briffa et al., 2002). Global warming has enhanced tree radial growth in cold‐humid regions (Babst et al., 2019), as rising temperatures have extended the growing season (Huang et al., 2010). However, an analysis of a global‐scale tree‐ring network revealed that the areas showing positive temperature effects on tree radial growth have decreased from 25.9% to 15.1% of tree growth area from 1930 to 1990, while areas showing positive precipitation effects remained unchanged and accounted for about 96%, and the areas showing negative VPD effects generally intensified from 82.0% to 92.8% (Babst et al., 2019). Similarly, the sensitivity of tree radial growth to precipitation has also increased since 1950 (Li et al., 2024). In addition, water limitation and future droughts are expected to suppress further growth and cause substantial declines in the radial growth of *Fagus sylvatica* (Martinez del Castillo et al., 2022; Klesse et al., 2024). These findings highlight that water limitation increasingly constrains tree radial growth under warmer and drier climates.

Water limitation is a major factor suppressing tree radial growth. Water deficit decreases cell turgor pressure, which in turn restricts cell division, expansion, and overall tree growth and development (Shao et al., 2008). Although global precipitation shows spatiotemporal heterogeneity across regions, the total global precipitation has remained relatively stable compared with the consistent rise in temperature (Yate and Ren, 2025). However, rising temperatures increase global atmospheric dryness and evapotranspiration, leading to drylands expanding to about 50% of the global land surface under future warming (Huang et al., 2016). Atmospheric dryness can be indicated by vapor pressure deficit (VPD), which is defined as the difference between the actual water vapor pressure and the saturation water vapor pressure (Nwayor et al., 2024). Rising temperatures increase the saturation vapor pressure, while the actual vapor pressure remains relatively stable, leading to an increase in global VPD, which exacerbates water shortage (Lian et al., 2021; Yuan et al., 2025). Global VPD has nearly doubled over the past century, with a significantly higher increasing trend observed in the past 20 years (Yuan et al., 2025). High VPD increases evaporative demand and induces stomatal closure to prevent excessive water loss, thereby reducing CO_2_ uptake and photosynthesis, ultimately constraining tree growth (Grossiord et al., 2020). In addition, elevated VPD can exacerbate soil water deficits and drought, increasing xylem tension and the risk of hydraulic embolism in trees (Grossiord et al., 2020). In western North America, a regional study based on a series of *Pseudotsuga menziesii* tree‐ring chronologies demonstrates that rising temperatures reduce radial growth by increasing VPD (Restaino et al., 2016). At the global scale, VPD has been shown to have contrasting effects on tree growth and forest productivity, driven by a threshold that separates positive from negative impacts (Zhong et al., 2023; Yuan et al., 2025). However, the specific contribution of VPD to tree radial growth is still not well quantified due to its interactions with other environmental factors (Yuan et al., 2025). Disentangling these effects is essential for improving our ability to model and predict tree growth under a future warmer and drier climate.

CO_2_ fertilization, resulting from rising atmospheric CO_2_ concentrations, increases carbon uptake and reduces stomatal conductance, thereby lowering tree water consumption and slowing soil moisture depletion, which in turn improves intrinsic water‐use efficiency (iWUE) and enhances photosynthesis (Huang et al., 2007; Keenan et al., 2013). This effect is considered a major driver of global vegetation greening (Piao et al., 2019b). Although the CO_2_ fertilization effect increases iWUE and may help mitigate water shortage, several studies indicate that its influence on tree radial growth remains limited (Peñuelas et al., 2011). A global tree‐ring study using stable carbon isotopes (δ^13^C) suggests a significant increase in iWUE (20.5%), while tree radial growth remained unchanged (Peñuelas et al., 2011). Similarly, in the tropics, a synthesis of tree‐ring δ^13^C data from 1,100 trees revealed a 30%–35% increase in iWUE, but no acceleration in tree radial growth (Van Der Sleen et al., 2015). These findings contrast with most dynamic global vegetation models, which simulate an increase in biomass under rising CO_2_ (Peñuelas et al., 2011; Van Der Sleen et al., 2015). The potential enhancement of tree radial growth by CO_2_ fertilization is likely constrained by climatic and biotic factors, such as temperature, drought, nutrient availability, and carbon allocation strategies (Peñuelas et al., 2011; Van Der Sleen et al., 2015). One possible explanation is that drought may shift carbon allocation from stems to belowground tissues, with more carbon invested in root growth to enhance water uptake rather than in wood formation (Ren et al., 2024). More data from both empirical and manipulated CO_2_ experiments are still needed to further investigate this discrepancy between rising atmospheric CO_2_ and the observed stability in tree radial growth.

### Tree radial growth response to climate extremes

Global warming has led to significant increases in daily hot records (four times higher), daily maximum precipitation records (40% higher), and monthly dryness records (10% higher) in the past decade compared with the scenario without climate change, resulting in more frequent, prolonged, and intense droughts, heatwaves, and floods (Fischer et al., 2025). Although overall cold events are decreasing, the extended growing season increases the risk of frosts (Liu et al., 2018). These climate extremes can substantially affect tree growth (Hartmann et al., 2022). However, the impacts of climate extremes on tree growth are difficult to quantify, and the mechanisms underlying these effects remain poorly understood. Reconstruction of disturbance history is therefore essential for assessing long‐term forest responses to extreme events and provides critical evidence for understanding how climate extremes affect tree growth (Altman, 2020). Tree rings provide ideal high‐resolution data for reconstructing disturbances in radial growth, and several methods have been developed to detect these disturbances (Altman, 2020; Wang et al., 2024a). However, even when potential disturbances are detected, attributing them to specific climate extremes remains challenging, as this typically requires detailed anatomical evidence, supporting literature, or local environmental conditions. A better understanding of how different climate extremes influence tree radial growth may enhance the modeling and prediction of the growth of trees and forests under climate change.

Drought has become one of the most widely studied climate extremes due to its strong disturbance to tree growth in recent decades. With ongoing global warming, the frequency, duration, and magnitude of droughts have significantly increased (AghaKouchak et al., 2020; Chen et al., 2025). Drought strongly suppresses tree radial growth and can even lead to mortality due to hydraulic failure or carbon starvation (Allen et al., 2010), thereby resulting in substantial impacts on forest ecosystems and the carbon cycle (Anderegg et al., 2015). Typically, drought induces immediate reductions in radial growth and produces legacy effects that can carry over into several subsequent years (Anderegg et al., 2015). Tree rings provide ideal data for capturing both the immediate and legacy effects of drought, as they are highly sensitive to water availability and record growth over long temporal scales (Kannenberg et al., 2020). Climatic water deficit, calculated as soil moisture minus potential evapotranspiration, has been shown to be the most effective indicator for capturing drought‐induced growth declines (Anderegg et al., 2015). Resistance (the ability of trees to maintain growth under drought) and resilience (the ability of trees to maintain growth under drought) are commonly used to quantify tree radial growth responses to drought (Li et al., 2020), enabling comparisons across species and regions (Schwarz et al., 2020). For example, a global tree‐ring width study found that the radial growth of gymnosperms shows higher drought resistance than that of angiosperms due to the former's conservative hydraulic and foliage traits (Li et al., 2020).

Following drought, the legacy effect of growth suppression is common. Evidence from tree‐ring records across diverse species and regions indicates that trees often show multi‐year growth lags in returning to pre‐drought growth following severe drought‐induced suppression (Anderegg et al., 2015; Kannenberg et al., 2020). Drought legacy effects can be attributed to a combination of delayed recovery in tree physiology (e.g., non‐structural carbohydrate depletion and hydraulic damage), post‐drought carbon allocation shifts (toward roots, canopy, or reproduction), and ecohydrological constraints such as slow replenishment of deep soil water (Kannenberg et al., 2020). Global tree‐ring analyses indicate that these legacy effects are most pronounced in arid regions and are substantially stronger in gymnosperms than in angiosperms (Anderegg et al., 2015).

Heatwaves have also become more frequent and often occur in combination with drought as compound events (Zscheischler et al., 2018). A heatwave is commonly defined as a period during which daily maximum temperatures exceed the 90th percentile of historical maximum temperatures (Perkins and Alexander, 2013; Salomón et al., 2022). Similar to drought, heatwaves induce stomatal closure to reduce transpiration, which in turn inhibits photosynthesis and decreases carbon uptake (Teskey et al., 2015). However, the impacts of heatwaves on tree radial growth show strong temporal variability. Dendrometer networks showed that the 2018 European heatwave caused pronounced stem dehydration and elevated tree water deficit, but annual radial growth did not decline consistently compared with the previous 2 years (Salomón et al., 2022). This limited impact on annual growth is likely because the heatwave occurred in July, when wood formation was almost completed (Salomón et al., 2022). Nevertheless, tree radial growth in different species shows diverse responses to heatwaves, and the potential legacy effects of heatwaves on radial growth remain unclear (Salomón et al., 2022).

Floods, caused by increasing intensity and frequency of heavy precipitation under climate change, can substantially affect tree radial growth (Ballesteros‐Cánovas et al., 2015). Several studies have used tree rings to reconstruct past flooding events (Wertz et al., 2013). Flooding can cause abnormal anatomical structures within the newly formed wood. Specifically, spring flooding in riparian forests can significantly reduce earlywood vessel size in submerged stem sections, resulting in flood rings (Copini et al., 2016). The flooding experiment showed that the mean earlywood vessel size in the flooded stem sections was reduced by about 50% compared to non‐flooded sections (Copini et al., 2016). The formation mechanisms of flood rings and their links to the timing and duration of flooding remain to be fully assessed. Floods can also affect tree radial growth through physical stress. Debris carried by floods can cause scars on tree stems (Stoffel and Bollschweiler, 2008). Floods can tilt trees through hydrodynamic pressure on the stem, and the resulting eccentric rings in the tree‐ring record can be used to date the flood event (Ballesteros‐Cánovas et al., 2015). Moreover, floods mainly occur in catchments and affect riparian forests in the floodplain (Ballesteros‐Cánovas et al., 2015), which limits the applicability of these studies to other regions and results in an insufficient understanding of the effects of extreme precipitation on tree radial growth.

Frost events have decreased overall due to global warming, but the extended growing season has led to a significant increase in frost days during the growing season across 43% of vegetated areas in the Northern Hemisphere (Liu et al., 2018). Frost events caused by cold air masses can damage leaves and reduce photosynthesis, or directly injure stems, thereby substantially reducing tree radial growth and overall productivity (Payette et al., 2010; Wang et al., 2025a). Newly unfolded leaves are highly vulnerable to late spring frost. Daily minimum temperature below −2.2°C after budburst is considered to cause severe damage to leaf tissues (Schwartz et al., 2006), thereby reducing photosynthetic capacity and suppressing radial growth (Wang et al., 2025a). Significant reductions in radial growth following late spring frost have been observed across the Northern Hemisphere (Wang et al., 2025a). In addition, the diverse responses of deciduous and evergreen trees to frost can help identify late spring frost events. Late spring frost damaged new leaves in deciduous species and suppressed radial growth, whereas evergreen conifers maintain photosynthesis through existing needles and therefore show no growth reduction under the same conditions (Vitasse et al., 2019, 2025). No significant legacy effects of late spring frost on radial growth have been observed in the following years (Vitasse et al., 2025). Moreover, frost can directly damage immature xylem cells, leaving a distinct anatomical record in tree rings known as frost rings. These are identified as rows of collapsed cells with poorly developed walls (Payette et al., 2010). The frost damage on xylem cells predominantly formed in the early growing season, as approximately 95% of the damage was identified within earlywood, and over 60% occurred in the first half of the earlywood (Payette et al., 2010). Notably, large volcanic eruptions can also trigger abrupt cooling events during the growing season that induce frost rings in trees on a global scale (Stoffel et al., 2015; Helama, 2023). Although growing‐season frost events are increasing under global warming and have significant impacts on tree radial growth, the extent to which they influence global forest growth and productivity has not yet been quantified.

## INTRA‐ANNUAL WOOD FORMATION RESPONSE TO CLIMATE CHANGE

Traditional dendrochronological studies use tree‐ring width as an indicator of tree growth over decadal to centennial or even millennial time scales, which can be applied to assess and predict long‐term forest growth dynamics under climate change (Wilmking et al., 2020). However, this approach neglects the influence of seasonal climatic factors on specific processes of xylem formation in tree stems (Rossi and Deslauriers, 2007). Despite the extensive dendrochronological records available, several unresolved issues remain in explaining the interannual variability of tree growth in response to environmental changes, such as the divergence problem and recently identified drought legacies (Peltier and Ogle, 2020). This is likely because tree growth dynamics and their responses to environmental drivers vary throughout the growing season. In this context, seasonal monitoring of wood formation (xylogenesis) provides precise, high‐resolution intra‐annual data on stem growth, offering fundamental insights into the mechanisms of tree‐ring formation and its linkage to climatic factors.

### Intra‐annual wood formation

Wood formation is a continuous and periodic process that occurs year to year, including four major sequential steps: Cell division, cell expansion and radial enlargement, cell wall thickening, and programmed cell death (Plomion et al., 2001). These processes originate from the activity of the vascular cambium, which consists of a few layers of cells with thin primary cell walls and narrow radial diameters, located between secondary xylem (i.e., the wood) and secondary phloem (i.e., the living bark). At the beginning of the growing seasons, when new cell plates are formed in the cambium zone (Begum et al., 2013), the vascular cambium begins to reactivate and produces phloem cells toward the bark and xylem cells toward the pith (Plomion et al., 2001). Newly formed xylem cells expand tangentially and radially by loosening primary cell walls (Cosgrove, 2005), driven by turgor pressure. Once cells reach their final size and expansion is complete, secondary cell wall formation begins. This cell wall thickening process involves the complex biosynthesis and deposition of cellulose, hemicellulose, cell wall proteins, and lignin, and is regulated by carbon supply (Deslauriers et al., 2016). When cell wall thickening is accomplished, programmed cell death occurs. During this process, the water‐conducting xylem cells mature and become fully functional by clearing all of the cellular contents through hydrolase activity (Häusser et al., 2023).

With the development of the micro‐sampling approach, the intra‐annual process of wood formation beneath the bark has become visible. This technique involves collecting tiny samples of wood (microcores, 2 mm in diameter and 3 cm in length) from the tree stem at weekly or biweekly intervals throughout the growing season using a tool called the Trephor (Rossi et al., 2006a), allowing researchers to observe developing wood cells at different stages. In this approach, high time resolution (weekly) and cell‐level data on cambium activity and wood formation are obtained, providing valuable insights into tree growth dynamics at a short time scale. Measurements of cambial and xylem activity provide critical insights into intra‐annual wood formation dynamics (Wang et al., 2024b), thereby improving our understanding of climatic factors (Huang et al., 2020, 2023; Wang et al., 2023a; Zhang et al., 2025) and the water and carbon allocation strategies governing tree growth at high temporal resolution (Martínez‐Sancho et al., 2022).

Over the past two decades, studies based on micro‐sampling have investigated cambial activity and xylem formation across global forests. In extratropical regions, cambium enters a dormant phase during winter and becomes active during spring and summer. In temperate and boreal forests, the dynamics of wood formation show S‐shaped curves, with the number of xylem cells rapidly increasing during late spring and early summer and reaching a plateau at the end of the growing season (Huang et al., 2014; Rossi et al., 2014). The growth rate follows a bell‐shaped curve, peaking around the summer solstice (Rossi et al., 2006b). In semi‐arid Mediterranean forests, wood cell production shows clear bimodal patterns, with growth peaking in the wet spring and autumn but halting in the dry summer (Camarero et al., 2010). In warm, humid subtropical forests, xylem formation is less affected by climatic factors but more closely associated with photosynthetically active radiation, and thus stems can grow continuously throughout the year (Huang et al., 2018). These contrasting patterns in cambial activity and xylem formation across different climate zones indicate the strong influence of regional climates on intra‐annual wood formation. Although temperature, water availability, and photoperiod have been documented as critical factors influencing wood formation, the timing, mechanisms, and extent to which these climatic factors affect the development of wood cells remain unclear.

### Climatic drivers of wood formation

#### Temperature

From field observations to experimental manipulations, temperature has been widely documented as the predominant factor triggering the onset of wood formation. Along 800 m of elevational gradient in the Swiss Alps, the onset of cell differentiation in *Larix decidua* was delayed 3–4 d per 100 m elevation upward (Moser et al., 2010). Similar results were recorded in the French Southern Alps, where the onset dates of cell enlargement, wall thickening, and maturation of *Larix decidua* showed a delay of 5.4, 5.2, and 3 d per °C increase, respectively (Saderi et al., 2019). A stem heating experiment further demonstrated that heat treatment induced earlier cambium reactivation of *Cryptomeria japonica*, and this response became stronger with heating lasting from winter to spring (Oribe and Kubo, 1997). Temperature may directly affect stem cambium cell division by influencing microtubule polymerization, a key cytoskeletal component that plays a critical role in cell division and differentiation (Begum et al., 2013; Rathgeber et al., 2016). In addition, temperature can indirectly regulate cambium activity by modulating hormone levels (Aloni, 2013; Ursache et al., 2013), which, in turn, affect the enzymatic activities essential for initiating and controlling the cell cycle (Stals and Inzé, 2001). Warmer temperatures can also facilitate sucrose allocation (Hoch and Körner, 2003) and stimulate growth‐related metabolic processes (Begum et al., 2007). As a result, rising temperatures may help meet the heat requirements for cell division, leading to earlier cambium reactivation and the start of wood cell production (Oribe and Kubo, 1997).

An open question is whether temperature initiates cambial activity and xylem formation once a critical threshold is reached, or through the accumulation of gradual warming. Rossi et al. (2007) identified a relatively stable temperature threshold between 5.6°C and 8.5°C for conifers growing in cold environments, above which xylem formation is initiated. Similar threshold effects have been reported in a timberline environment in southern Italy (Deslauriers et al., 2008), a dry inner Alpine valley (Swidrak et al., 2011), and the cold southeastern Tibetan Plateau (Li et al., 2017). Although the specific temperature thresholds vary among species and climate conditions (Begum et al., 2013), they tend to remain consistent within the same local environments. However, a recent study showed that chilling‐influenced heat‐sum models outperformed threshold models in predicting the onset of xylem cell enlargement, suggesting the ambivalent effects of temperature on spring wood phenology (Delpierre et al., 2019). Indeed, winter chilling and spring forcing represent the dual role of temperature in determining the timing of spring phenology of secondary growth (Huang et al., 2020), reflecting the safety mechanisms preventing tree growth at the wrong time (Beil et al., 2021). Similar to spring leaf phenology, endodormancy and ecodormancy were also observed in the periodic activity of the cambium zone in the stem, that is, the resting and quiescent stages (Begum et al., 2013). At the resting stage, the cambium cannot produce new xylem even under favorable environmental conditions. For example, localized heating of the stems of temperate *Cryptomeria japonica* in December did not induce reactivation of cambium (Begum et al., 2010). However, once the chilling requirements are reached, the cambium could respond to radial auxin concentration gradients (Begum et al., 2013) and then enter the dormant stages. At the end of dormancy, cambium becomes more responsive to warmer climates in spring (Körner and Hiltbrunner, 2018) and then regains the ability to produce new cells under appropriate environmental conditions (Begum et al., 2013). Warming‐induced insufficient winter chilling has been reported to slow down the advancement of spring leaf phenology (Fu et al., 2015), and this reduced chilling accumulation is often accompanied by increased heat requirements because chilling is negatively correlated with forcing (Harrington et al., 2010; Fu et al., 2013). However, it remains largely unclear how interactions between forcing and chilling affect spring wood phenology under climate warming. More efforts are needed to decipher the relative contributions of these two processes in regulating the timing of cambium activity and xylem formation, and their potential shifts under future warmer conditions.

For the process of cell wall thickening, Huang et al. (2023) reported the existence of a mean annual air temperature threshold of 4.9°C ± 1.1°C, based on a unique data set describing the onset of cell wall thickening in Northern Hemisphere conifers. This thermal threshold classified all the studied sites into cold and warm ecosystems, where mean annual air temperature and forcing, respectively, were the primary drivers for triggering the onset of cell wall thickening. In addition, day‐to‐day temperature variability in spring should also be considered as an important driver for wood formation and xylem phenology in future studies, because an increase in day‐to‐day spring temperature variability was found to explain the spatial synchrony in tree phenology increasingly reported across altitudes, latitudes, and urban–rural gradients (Zhang et al., 2024).

In contrast, there is no consensus on the influence of temperature on xylem growth cessation. Although large‐scale global syntheses have shown that the dates of the last enlarging cell and the wall‐thickening cell are delayed with increasing temperature (Rossi et al., 2016), this conclusion is contrary to observations at regional or local scales. For example, along elevational gradients in the Northern (Moser et al., 2010) and Southern Alps (Saderi et al., 2019), the cessation of wood formation in *Larix decidua* was found to be unrelated to altitude, suggesting minimal effects of temperatures on the end of cell differentiation. However, across the altitudinal gradient in the north‐western Himalaya, cell enlargement of *Abies pindrow* was completed about 3 weeks earlier at high elevations, whereas the termination of cell wall thickening showed little correlation with elevation (Malik et al., 2020). This implies that temperature plays an important role in regulating the cessation of cell enlargement but has a weaker influence on the termination of wall thickening. Cell enlargement and cell wall thickening are two distinct processes of wood formation and are dissociated at the cellular level (Cuny et al., 2015; Martínez–Sancho et al., 2022). The former contributes to the increased stem size, while the latter refers to woody biomass accumulation. This functional disconnect could partially explain the contrasting responses of these processes to temperature. The effect of temperature on the xylem growth might also be weakened by other factors such as soil nitrogen, because a wide spatial analysis from 75 conifer sites in the Northern Hemisphere found that rising soil nitrogen increases the delaying effects of elevated temperature on the end of xylem cell wall thickening but reduced the delaying effects on the cessation of cell enlargement, especially in humid regions (Zhang et al., 2025).

#### Photoperiod

Photoperiod is another important factor in regulating wood formation. A recent study compiling xylogenesis data from 21 coniferous species and spanning subtropical and boreal forests has shown that photoperiod also exerts a strong influence on the onset of wood formation (Huang et al., 2020). In addition, wood cell production was found to be synchronized with the annual photoperiod cycle (Cuny et al., 2015), with the peak timing of growth rates occurring at the summer solstice (Rossi et al., 2006b). Like leaf phenology, photoperiod also serves as a stable signal regulating wood formation. Shorter photoperiod prevents premature growth onset at early season, which may cause heavy damage during subsequent periods of late spring frost. The growth decline after the summer solstice allows trees to safely complete secondary cell wall formation and lignification (Rossi et al., 2006b) and investment in other organs and reserves (Körner et al., 2016; Etzold et al., 2022) before winter, thereby mitigating the increasing potential autumn frost risk under projected climate warming.

Photoperiod has also been associated with the cessation of wood formation (Micco et al., 2019). A synthesis study across the Northern Hemisphere indicated that photoperiod drives the cessation of xylem growth, and longer growing‐season photoperiod leads to earlier accomplishment of wood formation (Mu et al., 2023). A convergence in the onset of cell wall thickening toward the summer solstice was also revealed along the gradient of increase in temperature variability in the 75 sites over the northern hemisphere, indicating that trees can track to optimize carbon assimilation for cell wall thickening, as the most hours of sunlight are received around the summer solstice (Zhang et al., 2024). In addition, the observed similar ending dates of xylogenesis among years within the same region could imply the regulatory effects of photoperiod on xylem growth cessation (Deslauriers et al., 2008; Gruber et al., 2009). Nonetheless, caution should be exercised when accounting for photoperiod effects on growth dynamics, as photoperiod is closely tied to annual temperature variations. Although large‐scale and regional studies have emphasized the potential role of photoperiod in controlling tree phenology and growth dynamics, manipulative experiments are still needed to properly separate the effects of temperature from those of photoperiod (Partanen et al., 1998).

#### Water availability

Water conditions also play an important role in regulating the intra‐annual dynamics of wood formation, especially in relatively dry environments. Water availability is the premise of the onset of wood growth in arid and semi‐arid areas (Wang et al., 2023b). For example, the drier, rainless spring delayed the onset of xylem formation in *Juniperus przewalskii* on the northeastern Tibetan Plateau (Ren et al., 2015). Preseason soil water content was the main driver of cambial reactivation in *Pinus ponderosa* from the Mojave Desert Mountains, Southern Nevada (Ziaco et al., 2018). Water deficit was considered the potential trigger of wood growth cessation. For instance, a drier summer led to earlier cessation of xylem cell differentiation in *Juniperus przewalskii* on the Tibetan Plateau (Ren et al., 2015; Zhang et al., 2018). Across broad climatic gradients in South‐Western Germany, soil moisture showed a significant correlation with the date of xylem growth cessation (Miller et al., 2022). The rainfall exclusion experiment further indicated that lower soil moisture could lead to earlier termination of cell enlargement (Swidrak et al., 2013). Moreover, water conditions affect the amount of wood cell production. Higher precipitation contributed to a faster cell production rate in *Juniperus przewalskii* on the northeastern Tibetan Plateau (Ren et al., 2015) and in *Picea obovata* along the southern Altai Mountains (Wang et al., 2025b), thus promoting an increase in the production of wood cells at the end of growing seasons.

Indeed, water status is involved in several important processes of tree radial growth. In boreal forests, *Picea mariana* experiences a 40‐d springtime rehydration period before the growing season (Turcotte et al., 2009), which ensures that trees recover an adequate water balance after considerable winter water loss, and is a prerequisite for cambium cell division and xylem cell expansion. The available water could result from soil moisture, snowmelt (Castagneri et al., 2018), or precipitation (Shamir et al., 2020). In addition, the initiation of xylem growth, marked by the first appearance of enlarging cells, is a turgor‐driven process and highly sensitive to water conditions. Sufficient water availability enhances the flow of carbon compounds (Larysch et al., 2022), thereby helping maintain cellular osmotic balance and generating turgor pressure. Once cell turgor pressure exceeds a threshold (Lockhart, 1965), the primary cell wall loosens and expands, enabling irreversible cell division and expansion (Cosgrove, 2005). Recent studies confirmed that turgor‐driven cell enlargement is strongly associated with soil water potential. Tree stem growth initiated only when soil water potential reached a critical threshold of −0.6 MPa (Peters et al., 2021), and soil water potential determined the cessation timing of xylem cell production as well as the final number and size of tracheids (Cabon et al., 2020). As a result, intra‐annual wood formation responses to water shortage are extremely complicated. Long‐lasting and severe drought could slow down or even halt tree radial growth (Deslauriers et al., 2016), resulting in missing rings as observed in semi‐arid Northern China (Liang and Eckstein, 2006). Once water conditions improve, cambial activity resumes and produces early‐wood‐like cells after latewood formation (Vieira et al., 2017), resulting in a type of intra‐annual density fluctuations. This plastic response of wood formation to variability in water availability explains the bimodal patterns observed in the Mediterranean biome (Camarero et al., 2010), where two growth rate peaks matched well with spring and autumn rainfall periods.

## INTERACTIONS AMONG GROWTH PROCESSES

In addition to the direct effects of climate change and climate extremes on individual growth processes, such as canopy phenology, intra‐annual wood formation dynamics, and annual stem growth, the interactions among different growth processes play a critical role in determining overall tree growth (Figure 2), and these interactions may also be influenced by climate. Understanding these interactions is essential for evaluating how climate change influences the entire growth and forest productivity. However, these interactions and how climate affects them remain poorly understood.

### Leaf phenology–annual stem growth relationships

The timing of leaf unfolding and senescence not only reflects the developmental stages of leaves but also provides key indicators of the onset and cessation of the growing season in trees (Körner et al., 2023). Earlier leaf unfolding and later leaf senescence generally extend the growing season and the period of photosynthetic activity, promoting carbon assimilation (Keenan et al., 2014; Park et al., 2019) and increasing the carbon supply available for cambial activation and stem growth (Deslauriers et al., 2009). In addition, newly developed leaves release auxin (Schrader et al., 2003), which is transported to the stem, where it acts as a key signal triggering cambial reactivation and regulating wood formation (Aloni, 2013; Sorce et al., 2013; Guo et al., 2024), establishing a physiological link between leaf development and stem growth. As a result, an extended growing season, reflected by leaf phenology, is generally considered to enhance tree radial growth and forest productivity (Keenan et al., 2014).

However, several recent studies suggest that the relationship between canopy phenology and stem growth or productivity is generally weak. In North America, the SOS in temperate deciduous trees, as indicated by daily stem growth, advances with earlier canopy phenology derived from satellite observations (Dow et al., 2022). This earlier stem growth onset is often accompanied by an earlier cessation, resulting in no significant change in the total growth period and overall stem growth (Dow et al., 2022). Similarly, in Europe, a study combining *in situ*‐observed leaf phenology and tree‐ring data of deciduous trees has shown that, although rising temperatures advance leaf emergence and extend the growing season, such changes do not necessarily enhance radial growth or tree productivity (Camarero and Rubio‐Cuadrado, 2025). Furthermore, a study integrating leaf phenology, modeled wood formation, and tree‐ring data of conifers indicates a decoupling between leaf phenology and radial growth (Camarero et al., 2022). In addition to stem growth, neither the timing nor the length of the growing season determines productivity. An earlier SOS extends the growing season but reduces the mean daily carbon uptake rate across the Northern Hemisphere, diminishing its contribution to productivity, especially in arid regions (Liu et al., 2025c).

Although the growing season indicated by leaf phenology has started earlier and become longer in recent years, tree radial growth and productivity have shown no significant changes. This is likely because, while the growing season typically encompasses the period of stem growth, stem growth occurs discontinuously within this period (Körner et al., 2023). The analysis of daily stem growth measurements indicates that annual stem growth results from the accumulation of daily increments that occur only when climatic conditions are favorable for growth (Etzold et al., 2022). This suggests that the number of growth days, rather than the total length of the growing season, determines annual stem growth. Similarly, increases in forest productivity are driven primarily by higher mean daily carbon uptake rates rather than by a longer growing season (Liu et al., 2025c).

Sufficient observations of leaf phenology and tree radial growth are essential for investigating the relationship between the growing season and tree growth. Despite the extensive spatial coverage and the long time span of annual tree growth records, *in situ* observations of canopy phenology remain limited. Satellite‐derived phenological data only cover a relatively short period and may not capture the specific growth stages of trees due to the coarse spatial resolution. In addition to directly observed canopy phenology, the growing season can also be defined as the period during which meteorological conditions are suitable for plant growth (Körner et al., 2023). For example, the temperature threshold is the most used indicator for defining the SOS, as it is the primary climatic factor that initiates tree growth processes. A continuous period of 5 d with temperatures above 5°C is often defined as the thermal SOS (TSOS), which has been widely used in many studies as a proxy for the SOS instead of leaf phenology (Frich et al., 2002; Gao et al., 2022). By incorporating multiple indicators of the growing season, it is possible to establish a robust relationship between growing season and tree growth across extensive environmental gradients.

Studies investigating TSOS and tree stem growth over 60 years across the Northern Hemisphere have shown that an earlier TSOS enhances tree radial growth in humid regions but suppresses it in arid regions (Gao et al., 2022). Experimental manipulations indicate that the stem biomass in boreal forests increases with elevated temperature and ambient rainfall but decreases with higher temperature and reduced rainfall (Reich et al., 2022). Consequently, although rising temperatures lead to an earlier growing season, water availability plays a crucial role in regulating the relationship between phenology and tree growth. This is likely because an earlier growing season can exacerbate water shortage, particularly under dry conditions, and the subsequent water deficit may suppress all tree growth processes (Lian et al., 2020). However, the key drivers and the mechanisms underlying these relationships at a large scale remain poorly understood.

Most studies have focused on the canopy phenology–growth relationships within the same year, while the inter‐annual carry‐over effects have received much less attention. Changes in tree growth and productivity during the current year may influence canopy phenology in the following year. For instance, higher forest productivity has been associated with earlier leaf unfolding (SOS) in the subsequent spring (Gu et al., 2022). In contrast, climate extremes such as late spring frost or drought can delay SOS in the following spring by reducing photosynthetic capacity and carbon accumulation during the current growing season (Wang et al., 2025a; Liu et al., 2025c). Tree radial growth was found to significantly decrease after late spring frost across the Northern Hemisphere (Wang et al., 2025a). These complex patterns highlight the need for further research to clarify how canopy phenology–growth relationships affect tree growth and forest productivity across regions.

### Relationships between intra‐annual wood formation dynamics and annual stem growth and leaf phenology

The process of wood formation directly determines the total radial growth of tree stems, making it crucial to understand which stages of wood formation primarily control stem growth. Although climate warming has caused the earlier onset and longer duration of growing seasons for wood formation (Rossi et al., 2016; Huang et al., 2020), it remains uncertain whether these wood phenological shifts would translate into more wood growth and thus higher carbon sequestration. Studies from cold and humid boreal forests suggest that longer growing seasons have led to a disproportionate increase in xylem cell production (Rossi et al., 2014). However, this finding cannot be extrapolated to water‐limited forests, where growth rate rather than growing season length plays a decisive role in determining the final cell production (Ren et al., 2019). Conversely, longer growing seasons could even reduce cell production in these relatively dry areas by exacerbating water limitations during peak growing seasons (Wang et al., 2025b), introducing large uncertainties into woody biomass production and carbon sequestration. A recent study found that the cell production rate, rather than the onset and duration of cambium activity, determines wood growth (Wang et al., 2025b). While increasing evidence based on either tree‐ring analysis (Camarero et al., 2022; Gao et al., 2022) or dendrometer records (Dow et al., 2022; Bose et al., 2025) has shown that a longer growing season may not necessarily promote tree radial growth, the mechanism underlying it still needs to be clarified. Long‐term monitoring is therefore required to decipher how climate variations at finer temporal resolutions (daily or weekly) affect intra‐annual wood formation processes and to elucidate how these responses translate into long‐term radial growth trends under warmer, drier climate conditions.

Wood formation represents only one component of the complex physiological processes that trees integrate to determine whole‐tree growth under changing environmental conditions (Almagro et al., 2025). By linking the intra‐annual dynamic of wood formation with leaf development, Huang et al. (2014) found that xylem formation can be modeled as a function of cambium activity, bud phenology, and shoot and needle growth, contributing to the precise modeling of forest productivity and carbon cycling. Although efforts have been made to investigate the temporal coordination between primary and secondary growth, no consensus has been reached. The initiation of wood growth can occur before, after, or at the same time as budburst, depending on the year, site, and species (Rossi et al., 2009; Michelot et al., 2012; Dox et al., 2022). In *Pinus sylvestris*, wood formation begins before needle unfolding, indicating that the early radial growth is mainly derived from stored assimilates, such as non‐structural carbohydrates produced in the previous year (Michelot et al., 2012). In contrast, wood formation in *Fagus sylvatica* starts after budburst, suggesting that radial growth in this species is strongly dependent on leaf photosynthesis during the growing season (Michelot et al., 2012). In addition, phenological mismatches between above‐ and belowground growth have been observed (Liu et al., 2022a), highlighting the contrasting drivers of these two processes. A recent study compared wood growth of stems and coarse roots with leaf phenology in four common deciduous tree species in Western Europe and found that coarse root growth continued throughout the winter. In contrast, stem growth ceased in autumn (Marchand et al., 2025). This lack of seasonality in coarse root growth indicates that temperature has limited effects on belowground growth, and that intrinsic factors, such as carbohydrates or plant hormones, may exert greater influence on belowground processes. Previous studies have shown that trees often allocate more carbohydrates to leaves and roots during and after drought periods to restore canopy function and enhance water uptake (Doughty et al., 2015), often at the expense of stem growth. Under climate warming, understanding how stem growth is coordinated with the development of other organs and tissues throughout the growing season is essential for revealing whole‐tree carbon allocation strategies and adaptive responses to environmental stress.

## TREE GROWTH ADAPTATION TO CLIMATE CHANGE

**Figure 3:**
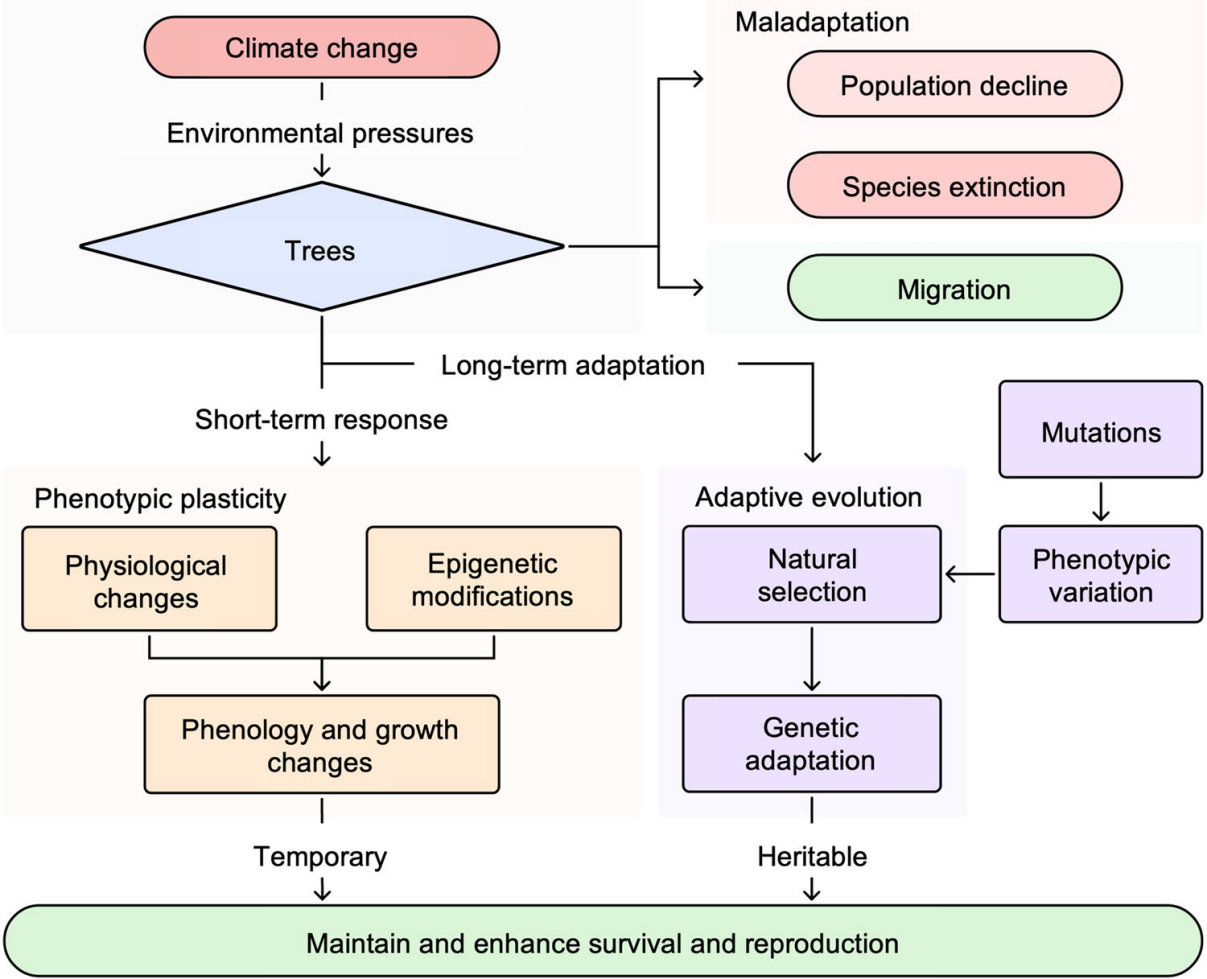
Flowchart of tree growth response and adaptation to climate change Trees respond to rapid climate fluctuations through short‐term physiological and phenological changes, whereas prolonged environmental changes can lead to long‐term genetic adaptations under natural selection, ultimately maintaining and enhancing tree survival and reproduction. Trees may migrate to more suitable habitats, resulting in shifts in their distribution range. Failure to adapt can lead to maladaptation, resulting in increased individual mortality, population decline, and potentially species extinction.

Adaptation is defined as the process of evolutionary change (e.g., natural selection) that results in phenotypic traits that enhance survival and reproduction in organisms (i.e., increase Darwinian fitness) (Figure 3; Rose and Burke, 2013; Yona et al., 2015). Adaptation consists of a series of processes of heritable genetic changes over multiple time scales, including epigenetic modifications and mutations (Yona et al., 2015). Tree growth initially responds to climate change through rapid physiological adjustments, such as changes in canopy phenology and stem growth. Over longer time scales, these short‐term responses may contribute to long‐term shifts in traits that enhance growth and reproduction in new climatic conditions under natural selection. The following section focuses on molecular aspects, discussing the short‐term physiological adjustments and long‐term genetic adaptations of tree growth under a changing climate.

### Physiological and epigenetic modifications

Physiological and epigenetic modifications involve the activation of specific genes and changes in gene expression that enable trees to cope with climate change, but they are not heritable. Leaf development is governed by genetic networks that control cell growth, differentiation, and responses to intrinsic programs and environmental signals. The *KNOTTED1‐LIKE HOMEOBOX* (*KNOX*) maintains meristem activity and integrates hormonal signals such as auxin and cytokinin to regulate leaf development, which has been comprehensively reviewed (Jia et al., 2023). Leaf phenology is similarly regulated by multiple genetic drivers, whose expression can be altered by climate change. Short photoperiods activate *FLOWERING LOCUS D1* (*FLD1*) and *ABSCISIC ACID INSENSITIVE 3* (*ABI3*) to promote bud set, while increased exposure to chilling reduces the expression of growth repressors such as *CENTRORADIALIS‐LIKE1* (*CENL1*) to break endodormancy, and long days with warm temperatures subsequently activate factors such as *FLOWERING LOCUS T1* (*FT1*) to release ecodormancy and initiate budburst (Maurya et al., 2018).

Temperature significantly impacts the gene expression related to wood formation in trees. For example, in *Eucalyptus* spp. exposed to temperature gradients, it was revealed that relatively high temperatures (30°C–35°C) profoundly change gene expression in the stem, primarily through the upregulation of genes involved in carbon metabolism, cell wall formation, and hormonal regulation (Feltrim et al., 2022). Such adjustments reflect the molecular basis of thermal adaptation in trees. In addition, multiple regulators, including hormones, peptides, and transcription factors, act to control wood formation. The detailed signaling pathways and molecular regulators underlying wood formation have been comprehensively reviewed (Zhu and Li, 2024). A better understanding of these climate‐induced genetic changes is crucial for developing effective strategies to mitigate the effects of climate change on forest ecosystems and ensure sustainable wood production.

Genome‐wide association studies (GWAS) have further revealed numerous genomic loci underlying variation in leaf and wood traits, uncovering the core genetic networks that govern growth adaptation to climate change. For instance, genes associated with leaf unfolding and senescence in *Fagus sylvatica* have been found to be primarily linked to circadian rhythm and dormancy pathways (Pfenninger et al., 2025). Specifically, eight genes were associated with circadian regulation and leaf unfolding, and nine genes were associated with fall dormancy or leaf senescence (Pfenninger et al., 2025). These genetic results also enabled the development of accurate models to predict leaf phenology, highlighting the potential of combining observation with genomic analysis to assess phenological variation (Pfenninger et al., 2025). In addition, based on increment cores from young *Populus trichocarpa* trees in a common garden, 141 significant single‐nucleotide polymorphisms (SNPs) were identified to be associated with 16 wood chemistry and structural traits, contributing to phenotypic variation in wood (Porth et al., 2013). In a common garden experiment, stomatal traits and genes were measured from leaf tissue of transplanted *Populus trichocarpa* cuttings to investigate genetic adaptation to drought (Klein et al., 2025). In arid regions, stomata tend to be smaller, and key loci influencing stomatal size have been identified, contributing to adaptation to drought (Klein et al., 2025).

Epigenetic modifications, such as DNA methylation, also play a critical role in enabling trees to respond to climate variability without altering the nucleotide sequence (Gutzat and Mittelsten Scheid, 2012; Sun et al., 2022). For instance, the DNA methylation level of *Populus trichocarpa* is influenced by temperature and precipitation, which in turn regulates the expression of the resin synthesis‐related transcription factor *BASIC HELIX‐LOOP‐HELIX 9* (*bHLH9*) (Wang et al., 2023c). Under high temperatures or low precipitation, reduced methylation leads to the upregulation of *bHLH9*, promoting resin production and aiding trees in resisting stresses such as intense light and drought (Wang et al., 2023c). Such epigenetic regulation not only enables trees to respond to environmental stress, but some stress‐induced epigenetic marks can also be partially maintained and transmitted through asexual reproduction, potentially influencing offspring phenotypes (Vanden Broeck et al., 2018).

However, most of these studies were conducted on seedlings or annual plants in common‐garden experiments, and how mature, long‐lived trees adapt their growth under natural forest conditions remains largely unexplored. Common‐garden experiments have several limitations. Most studies only focus on a limited set of environmental conditions, whereas tree growth in nature is influenced by complex climatic variation with climate extremes, which are difficult to simulate under experiments. In addition, common‐garden studies typically focus on seedlings, but their climate sensitivity may not reflect the sensitivity of long‐lived species, as individual development itself can also be influenced by climate adaptation (Schwinning et al., 2022). Observation of adaptation over an entire life cycle is particularly challenging for long‐lived species in short‐term experiments (Schwinning et al., 2022). Accelerated growth has been shown to increase the rate of epigenetic changes in tree stems (Zhou et al., 2025). Because mature trees typically show slower and more stable growth in forests, they may experience lower rates of epigenetic changes compared with seedlings. As a result, studies based on seedlings in a common garden may overestimate the capacity for epigenetic adaptation in tree growth to climate change compared to natural populations.

Conducting studies on mature trees under natural forest conditions is essential for understanding how tree growth adapts to climate change. To address this, our study focused on natural populations of *Larix sibirica* in the southern Altai Mountains of northwestern China. We collected coniferous leaf tissues from 12 sites, with three trees at each site, and generated leaf transcriptomes. Temperature was identified as the key climatic factor regulating the expression of multiple gene modules (Liu et al., 2022b). Recently, our unpublished study combined tree‐ring width data and gene expression to further explore the interactions among phenotype, gene, and climate. We integrated gene expression data from needleleaf trees with climate information to model 30 years of tree‐ring width in Larix sibirica across 12 sites. Needleleaf samples were chosen because they reflect changes in gene expression related to photosynthesis, energy metabolism, and stress response. These physiological processes directly influence tree growth and the accumulation of metabolic products, which in turn indirectly affect cambial activity. Climatic factors such as summer precipitation and diurnal temperature range can influence a gene co‐expression module, which in turn affects tree radial growth. These results indicate that gene expression serves as a crucial link between climate and phenotype. This study provides new insights into how mature trees adapt to climate change in natural forests and highlights the potential of incorporating gene expression data into radial growth modeling.

### Evolutionary adaptation and migration

Changes in DNA copy number and genetic mutations can modify not only gene expression but also the protein sequence and function, providing more durable adaptations under selective environmental pressures (Yona et al., 2015). Increasing temperatures from 23°C to 29°C can induce the accumulation of single‐nucleotide variants in *Arabidopsis thaliana* genomes, suggesting that genetic mutation rates may increase under stress conditions (Belfield et al., 2021). The flowering time of *Brassica rapa* seeds after drought is earlier than that of stored seeds before the drought, indicating that natural selection for drought can drive adaptive evolution within just a few generations (Franks et al., 2007). However, most observations of genetic adaptation come from common‐garden experiments using seedlings or annual plants, while evidence from mature, long‐lived trees under natural conditions remains scarce. Because these heritable adaptations take a long time to be expressed, it remains largely unclear how such durable adaptations enable trees to cope with climate change.

Genetic adaptation typically operates over long time scales. Studies of tree evolution over million‐year periods provide evidence of their capacity to adapt to past climate changes. For example, *Callitris* in Australia diversified with the onset of aridity in the early Oligocene (30 million years ago), evolving extreme embolism resistance in its xylem that enabled drought adaptation and successful expansion into arid environments (Larter et al., 2017). However, due to their long generation times, the pace of evolutionary adaptation in trees may lag behind the rapid rate of climate change, leaving them particularly vulnerable and warranting special consideration (Aitken et al., 2008). Trees show rich genetic diversity and extensive gene flow (e.g., via pollen and seed dispersal), which provides the potential for adaptation and evolution across different climatic gradients. Indeed, common European tree species were able to preserve their genetic diversity during periods of major climatic change, despite pronounced reductions in population size and shifts in distribution during glacial cycles (Milesi et al., 2024).

At the population level, if trees are unable to adapt to climate change through physiological adjustments or if new areas become available for colonization, trees may migrate to adjust their geographic distribution to more suitable environments. Past glacial and postglacial migrations, revealed from fossil pollen, demonstrate a strong capacity for range shifts (Aitken et al., 2008). Paleo‐distribution modeling of 25 North American tree species from 20 to 5 thousand years ago (ka) revealed migration trajectories and rates, consistent with pollen records (Hanberry, 2023), providing a clear understanding of past distributions and movements that reflect tree adaptations to climate change. At around 20 ka (Last Glacial Maximum), the distribution of *Picea mariana* was limited to the northern edge of the ice sheet (about 50°N) in North America due to cooling and extensive glaciation (Hanberry, 2023). Following the post‐glacial warming and deglaciation, the species ultimately reached its modern northern limit of approximately 70°N. However, the northward migration rates varied over time, approximately 5 km per century between 20 and 14 ka, 19 km per century between 14 and 10 ka, and 4 km per century between 10 and 5 ka, reflecting changes in the rate of warming (Hanberry, 2023). The future distributions of several European native tree species were also projected under climate change. Conifers such as *Abies alba* and *Picea abies*, projected to lose over 50% of their current climatic suitability, are considered the most threatened under climate change, whereas broadleaf species like *Ulmus minor* and *Acer pseudoplatanus* are relatively less threatened (Dyderski et al., 2025). Forest composition is likely to shift from coniferous to broadleaved species, reflecting the competitive traits of broadleaves, such as larger leaves and lower wood density, which may make them more adaptable under climate change (Dyderski et al., 2025). These predictions provide valuable guidance for conservation and management strategies in forests. However, recent models based solely on climate should be improved by incorporating additional environmental factors, such as soil properties, to enhance their reliability (Dyderski et al., 2025).

Although trees generally have intrinsic adaptive capacity and mitigation potential, projected changes in temperature and precipitation are likely to disrupt tree adaptation and natural migration, which may limit local growth and decrease carbon stocks (Frank et al., 2017; Gustafson et al., 2023). Assisted migration can facilitate tree adaptation to climate change and help maintain the resilience of forests and their ecosystem functions (Gustafson et al., 2023). Species distribution models project that conifers will become largely unsuitable for growth across Europe and are likely to be replaced by deciduous species (Chakraborty et al., 2024). Particularly, under Representative Concentration Pathways (RCP) 8.5 during 2061–2080, the suitable potential cultivation area of *Abies alba* is projected to decline by nearly 100%, whereas that of *Quercus robur* may increase by 600% (Chakraborty et al., 2024). Therefore, assisted migration of deciduous species is recommended to replace coniferous forests in order to enhance forest resilience and increase carbon storage (Chakraborty et al., 2024). These predictions included only a limited number of tree species and did not account for climate extremes, which limits their generalizability and ability to capture variability (Chakraborty et al., 2024).

## PANTROPICAL TREE GROWTH RESPONSE TO CLIMATE CHANGE

Most research on tree growth responses and adaptations to climate change has been conducted in temperate and boreal forests, whereas studies in tropical forests remain limited. In this section, we specifically focus on the tropical forests for the purpose of attracting more attention in future research. Tropical forests contain the largest carbon stocks across global forest ecosystems, accounting for about 55% of global forest biomass and showing the highest rates of carbon stock change (Pan et al., 2011), which play a critical role in regulating global water and carbon cycles (Mitchard, 2018). Tropical regions are experiencing rapidly increasing temperatures and declining precipitation under climate change, which have substantially affected tropical forest growth (Locosselli et al., 2020; Aguirre‐Gutiérrez et al., 2025). However, limited studies on tree growth responses to climate change in tropical regions, compared with temperate and boreal forests (Sheldon, 2019; Groenendijk et al., 2025), are largely due to the scarcity of long‐term observations, sampling data, and reliable meteorological records (Harris et al., 2020; Groenendijk et al., 2025). In addition, in most tropical regions, the seasons are not clearly defined, and many tropical tree species lack a distinct annual rhythm between growth and dormancy (Goldstein and Santiago, 2016), which represents a challenge for investigation of seasonal changes in tree growth responses to climate change. Therefore, understanding tree growth in tropical regions is essential for accurately modeling and predicting global forest responses to future climate change (Mitchard, 2018).

Tropical canopy phenology is highly complex and shows considerable variations due to the high diversity of species (Staggemeier et al., 2020). In tropical regions, growing seasons often span the entire year, and intra‐ and inter‐annual rainfall cycles or cloud‐induced variations in solar radiation may serve as regulators of phenology, rather than temperature and photoperiod (Davis et al., 2022; Terasaki Hart et al., 2025). Studies on the phenological responses of tropical trees to climate change remain scarce, and the drivers of tropical phenology are still poorly understood (Park et al., 2021; Davis et al., 2022). The limited availability of observational data on tropical canopy phenology constrains our understanding of tropical phenology. Although large‐scale remote sensing has been used to derive tropical canopy phenology (Moore et al., 2017), observations at the individual‐tree level remain sparse (Abernethy et al., 2018). This suggests that additional field observations are needed, and herbarium specimens could be used to extend phenological records (Davis et al., 2022). In addition, incorporation of phylogenetic signals may improve models of tropical phenology (Davis et al., 2022), which has been demonstrated to enhance estimates of flowering in tropical forests (Staggemeier et al., 2020).

Tree‐ring studies are extremely rare in tropical and subtropical regions, as only a limited number of tree species can form distinct annual rings in these regions (Groenendijk et al., 2025). Nevertheless, tree‐ring analyses in tropical regions can provide valuable insights into the long‐term radial growth responses to climate change. In addition, tree‐ring studies have good potential for use for the reconstruction of past climate to fill the gaps in meteorological records in tropical regions (Groenendijk et al., 2025). Tree‐ring studies in southeastern subtropical China suggest that competition rather than climate plays a critical role in regulating tree radial growth (Liang et al., 2019). Recently, a tree‐ring data set has been developed, containing about 500 chronologies derived from over 20,000 individual ring‐width series from about 10,000 trees representing 139 tree species across pantropical regions (30°N–30°S) (Groenendijk et al., 2025). Although a growing number of studies have utilized these data to investigate tropical tree growth, how tropical trees respond to climate change remains unclear. Variations in tropical tree growth have been shown to be driven by climate in the dry season (Zuidema et al., 2022), and the longevity of tropical trees may decline under warmer and drier conditions due to increased evaporation and associated mortality (Locosselli et al., 2020). However, other studies have found that drought has a limited influence on stem growth, as its negative impacts can be offset by growth stimulation during the wet season (Zuidema et al., 2025). Furthermore, studies in Africa and in extreme arid and humid regions are scarce, which may limit the representativeness of research on tropical tree growth responses to climate change (Zuidema et al., 2025). A map of potential sites suitable for tree‐ring research in tropical and subtropical regions has been developed, which identifies regions with tree species that can form tree rings (Groenendijk et al., 2025), thereby encouraging further tree‐ring study in these tropical and subtropical regions.

Intra‐annual wood formation studies conducted in tropical and subtropical forests are fewer than those in temperate and boreal regions. Most studies in subtropical regions have focused on conifers due to their relatively simple, well‐defined wood structure. For example, in subtropical South China, studies of wood formation indicate that *Pinus massoniana* shows relatively high growth rates during the dry autumn, suggesting a different growing season compared with temperate and boreal forests (Huang et al., 2018). Investigations on broadleaf hardwood species remain limited, as these trees possess more complex hydraulic architectures and variable wood porosity. Such efforts are crucial for filling existing knowledge gaps and for developing more accurate models to predict forest productivity and carbon dynamics at global scales.

## FUTURE DIRECTIONS IN MODELING AND PREDICTING TREE GROWTH

Tree growth responses and adaptations to climate change are complex and highly variable due to regional variations in climate and the diverse sensitivities of tree species to different climatic factors. Further research is needed to improve our understanding of these responses and the underlying mechanisms, including studies on canopy phenology, intra‐annual wood formation dynamics, tree radial growth interactions among growth processes, and genetic adaptation, supported by integrative sampling strategies with corresponding high spatial and temporal resolution climate observations, which are essential for enhancing tree growth modeling and prediction.

Future research on canopy phenology should examine whether continued winter warming and reduced chilling accumulation may slow or reverse advances in spring leaf phenology. Phenology models need to incorporate water availability to better model and predict both the timing of leaf unfolding and senescence. Greater emphasis on leaf senescence is required to improve predictions of leaf‐based growing‐season length and forest carbon sequestration under future climate warming.

For tree radial growth, efforts should focus on quantifying how increasing water limitation modifies growth responses to warming and CO_2_ fertilization at the global scale. Improving understanding of climate extremes and integrating their impacts into growth models will be essential for enhancing projections of forest productivity and carbon equilibrium under future climate warming.

Monitoring of intra‐annual wood formation dynamics is crucial to understanding how climate regulates stem growth at fine temporal resolution. Future studies should aim to disentangle the combined role of temperature, including chilling and forcing, and photoperiod in regulating cambium activity and xylem formation. The relative contributions of temperature accumulation compared with threshold‐based temperature to the onset of wood formation remain unclear. Water availability is another critical factor influencing the onset, cessation, and rate of wood formation, especially in relatively dry regions. The key thresholds of water availability that regulate cambial and xylem activity over the global scale should be quantified.

The interactions among leaf phenology, intra‐annual wood formation dynamics, and overall stem growth are complex and remain poorly understood. Future studies should quantify the extent to which leaf‐ and wood‐based growing seasons determine annual stem growth and forest productivity. The carry‐over effects of tree radial growth on leaf phenology and intra‐annual wood formation dynamics in the following year are still largely unknown. How climate change influences these interactions also remains unclear. Although interactions between leaf phenology and xylem formation can strongly affect each growth process, disentangling these effects from the direct impacts of climate remains a major challenge.

Gene expression serves as a crucial link between physiological processes and climatic drivers, regulating short‐term responses of tree growth to climate fluctuations. Most research on tree growth has primarily focused on tree phenotypic changes in response to climate change, while paying less attention to the underlying gene regulatory mechanisms. Understanding the processes of genetic change and identifying the specific genes involved are crucial for revealing the mechanisms underlying tree growth adaptation to rapidly changing environmental conditions. However, most studies have been conducted on seedlings or annual plants in common‐garden experiments, whereas investigations of mature trees in their natural habitats, which better represent tree growth adaptation to climate change, remain scarce. Furthermore, the long‐term heritable adaptations in tree growth under climate change remain largely unclear.

Long‐term monitoring of intra‐annual wood formation is scarce, as micro‐sampling approaches are highly time‐consuming and labor‐intensive, involving frequent field sampling, complex laboratory procedures, and microscopic analyses across multiple growing seasons. Therefore, greater efforts are needed to broaden the research in intra‐annual wood formation across diverse functional groups and climate zones over long periods, with particular emphasis on broadleaf species in tropical and subtropical forests. Establishment of long‐term monitoring networks would substantially advance our understanding of tree growth processes and their responses to changing climates.

Integrative observations across different tree organs and growth processes are essential for a comprehensive understanding of whole tree growth responses to climate change. Although a large number of phenology and tree‐ring data sets are available, much of the data were collected at different sites and times, resulting in the studies on different processes of tree growth response to climate change being disconnected. This makes it difficult to systematically study the multiple aspects of tree growth and their interactions under climate change. Integrative *in situ* observations and increased sampling efforts, especially in tropical and subtropical regions, are encouraged to enhance understanding of the interactions among multiple growth processes.

Various climate data sets with differing spatial and temporal resolutions are available. Selecting the most appropriate climate data sets is crucial. Studies have shown that climate data with higher temporal and spatial resolution and microclimate better capture detailed climate variability and improve the modeling of tree growth (Klinges et al., 2024; Yang et al., 2025). In addition to using open‐access climate data, we also suggest establishing a meteorological station within 500 m of the study sites to obtain the most accurate vertical climate profile from the ground to the canopy for modeling the growth of different tree organs.

The assumptions underlying predictions of tree growth response to climate change have limitations. The space‐for‐time substitution strategy is often used to predict future tree growth by using a large spatial climate gradient to fill the gap of long‐term observation (Evans et al., 2025). The reliability of this method has been questioned, as it may cause significant errors in the magnitude and direction of the effect of climate change on tree growth. This is because the relationships inferred from regression analyses are not necessarily causal, as tree growth can be influenced by multiple factors other than climate (Evans et al., 2025). Therefore, it is important to acknowledge these limitations and interpret regression‐based results with caution. In addition, most tree growth models assume that the relationship between growth and climate remains constant over time. However, the sensitivity of tree growth to climate can change (D'Arrigo et al., 2008). Under substantial climate change, trees may be exposed to climatic conditions that they have never experienced before (Aguirre‐Gutiérrez et al., 2025), and tree growth response may mismatch or lag behind the pace of climate change (Evans et al., 2025). To improve predictions, more observational, sampling, and experimental data are needed to investigate the dynamic relationship between tree growth and climate (Hänninen et al., 2019; Evans et al., 2025). Additionally, development of process‐based models can strengthen mechanistic understanding, clarify the causal relationship between tree growth and climate, and enhance the reliability of predictions (Evans et al., 2025).

To improve our understanding of tree growth response and adaptation to climate change, multidisciplinary integration across multiple scales is required. At site scales, integration of phenology, wood anatomy, and physiology can reveal how different tree organs and growth processes coordinate and how resources are allocated to regulate growth processes under climate change. At regional scales, linking field observations with remote sensing and climate data helps identify spatial patterns and long‐term trends in tree growth. At broader conceptual levels, integration of ecological, climatic, and evolutionary perspectives is crucial for predicting how forests adapt to future climate change. Such multi‐level, cross‐disciplinary approaches will improve our ability to model global forest dynamics and carbon cycling and support the development of effective forest management strategies to enhance carbon sequestration and mitigate global warming.

## CONFLICTS OF INTEREST

The authors declare no conflicts of interest.

## AUTHOR CONTRIBUTIONS

J.G.H. structured and supervised the review. F.Y., L.Z., J.C., F.Y., and W.W. wrote the manuscript. J.G.H., H.L., Q.M., and B.C. reviewed and edited the manuscript. All authors have read and approved the contents of this paper.
